# New Vistas for the Relationship between Empathy and Political Ideology

**DOI:** 10.1523/ENEURO.0086-24.2024

**Published:** 2024-11-15

**Authors:** Niloufar Zebarjadi, Annika Kluge, Eliyahu Adler, Jonathan Levy

**Affiliations:** ^1^Department of Neuroscience and Biomedical Engineering, Aalto University, Espoo 02150, Finland; ^2^Department of Psychology, The Hebrew University of Jerusalem, Jerusalem 91905, Israel; ^3^Department of Criminology and Gonda Brain Research Center, Bar Ilan University, Ramat-Gan 5290002, Israel

**Keywords:** empathy, neuroimaging, pain empathy, political ideology

## Abstract

The study of ideological asymmetries in empathy has consistently yielded inconclusive findings. Yet, until recently these inconsistencies relied exclusively on self-reports, which are known to be prone to biases and inaccuracies when evaluating empathy levels. Very recently, we reported ideological asymmetries in cognitive-affective empathy while relying on neuroimaging for the first time to address this question. In the present investigation which sampled a large cohort of human individuals from two distant countries and neuroimaging sites, we re-examine this question, but this time from the perspective of empathy to physical pain. The results are unambiguous at the neural and behavioral levels and showcase no asymmetry. This finding raises a novel premise: the question of whether empathy is ideologically asymmetrical depends on the targeted component of empathy (e.g., physical pain vs cognitive-affective) and requires explicit but also unobtrusive techniques for the measure of empathy. Moreover, the findings shed new light on another line of research investigating ideological (a)symmetries in physiological responses to vicarious pain, disgust, and threat.

## Significance Statement

In this study, we challenge the historically inconclusive findings on ideological asymmetries in empathy. By employing neuroimaging techniques, we demonstrated that ideological asymmetry in physical pain empathy is absent. This research underscores the importance of considering various facets of empathy, societal contexts, and unbiased measurement methodologies, such as neuroimaging techniques, in the assessment of empathic responses.

## Introduction

The growing body of literature on the interplay between political ideologies and psychophysiological processes reveals mixed findings (Wagaman and Segal, 2014; Waytz et al., 2016; Hasson et al., 2018; Morris, 2020; Smith and Warren, 2020). A number of these studies have proposed the existence of potential biopsychological underpinnings to political ideologies. For instance, to examine possible ideological differences in levels of empathy, several researchers reported enhanced empathy among “leftists” (i.e., ideological left, or liberals) compared with “rightists” (i.e., ideological right, or conservatives; Wagaman and Segal, 2014; Hasson et al., 2018; Morris, 2020). However, other studies failed to reproduce those findings (Waytz et al., 2016) or even reported reversed patterns (Casey et al., 2023). A potential factor contributing to this inconsistency might be the monolithic definition of the term “empathy,” often overlooking context and nuanced distinctions. Recently, it has been proposed that empathy researchers should relate to empathy as a multifaceted social-cognitive phenomenon, encompassing diverse components, such as physical pain, affect, mentalization, and empathic care (Lamm et al., 2007; Weisz and Cikara, 2021). The involvement of each component may vary depending on how empathy is triggered during the experimental procedures (Lamm et al., 2011; Bruneau et al., 2012), highlighting the importance of considering the type of stimuli, context, and individual factors.

An alternative explanation for the heterogeneous findings is the methodology for assessing empathy. To date, the interplay between empathy and political ideology has been predominantly examined using self-reports (Pliskin et al., 2014; Wagaman and Segal, 2014; Hasson et al., 2018; Morris, 2020). Yet, various empathy scholars have unraveled the benefits and promises of the neuroscientific perspective on the multiple facets of empathy (Zaki and Ochsner, 2012; Schreiber, 2017; Weisz and Cikara, 2021; Zebarjadi et al., 2023). Very recently, the first neuroimaging study explored the relationship between political ideology and affective-cognitive empathy (Zebarjadi et al., 2023). In that study, a brief story of characters was depicted, followed by displaying several distressing emotional or neutral pictures of them. This sort of stimulation requires participants to mentalize with the characters of the stories throughout the assessment. Brain activation during this task was localized in the temporal parietal junction (TPJ), an area associated with perspective-taking and the cognitive facet of empathy. This study revealed that compared with left-wing, right-wing ideological values are associated with reduced neural underpinnings of affective-cognitive empathy.

Although that study was valuable for consolidating the relationship between political ideology and empathy, it remains underexplored whether this relationship might depend on the subprocesses of empathy (Weisz and Cikara, 2021). In particular, although previous studies reported ideological asymmetry in relation to cognitive and affective facets of empathy (Wagaman and Segal, 2014; Hasson et al., 2018; Morris, 2020; Casey et al., 2023; Zebarjadi et al., 2023), it was rarely examined whether empathy to vicarious physical pain is also ideologically sensitive. The debate on ideological asymmetry largely centered on the neural and physiological responses to images depicting vicarious physical pain, threat, and disgust (Oxley et al., 2008; Ahn et al., 2014; Bakker et al., 2020; Brandt and Bakker, 2022), with Oxley et al. (2008) being one of the pioneering studies. They suggested that individuals with higher physiological reactivity to threatening stimuli were more likely to endorse conservative policies, whereas those with lower sensitivity leaned toward liberal stances. However, subsequent studies found conflicting evidence, with Bakker et al. (2020) failing to replicate these results and Brandt et al. (2014b) arguing that both conservatives and liberals may react similarly to threatening stimuli (Brandt et al., 2014b; Bakker et al., 2020; Brandt & Bakker, 2022). This ongoing debate emphasizes the need for further exploration and more accurate methods to understand the interplay between psychophysiological traits and political inclinations (Smith and Warren, 2020).

Building on those recent findings, we extend our investigation to examine whether empathy toward vicarious physical distress and pain is ideologically independent (Zebarjadi et al., 2023). To increase the reliability of the study, we employ two important strategies that have been neglected in the past literature: First, neuroimaging (MEG) is implemented in conjunction with various behavioral and self-reported measures of empathy. Many past MEG studies proved that empathy toward vicarious physical pain and suffering triggers a clear and robust neural response (Perry et al., 2010; Whitmarsh et al., 2011; Motoyama et al., 2017; Levy et al., 2018; Zebarjadi et al., 2021; Zebarjadi and Levy, 2023). These studies indicated that the neural mechanism underlying empathy mainly reported the involvement of the alpha rhythm. Besides, other studies pointed out a positive correlation between the suppression of alpha-band power and functional activation in a specific brain region (Jensen and Mazaheri, 2010; Schubring and Schupp, 2021). Therefore, based on the robust findings in the previous MEG studies on empathy, in the current study, we examined the neural response within the alpha-band range. Second, a large neuroimaging sample was used in two different continents and experimental sites to increase the generalizability of findings.

## Materials and Methods

### Study 1

#### Participants

Seventy-seven healthy Jewish Israeli participants (36 females; mean age ± SD, 25.3 ± 3.83, 46 politically rightist and 31 politically leftist) participated in this study. Before the recruitment, subjects were assessed for MEG compatibility as well as their psychiatric and neurological history. In addition, participants were asked to report their demographics (such as gender, and age) and political ideology. All instructions were delivered in the participants’ native language, and they were remunerated for their participation in the study. The ethics committee at IDC Herzliya approved the study, and all participants signed the consent form.

#### Stimuli

A set of well-validated stimuli, similar to our previous experiments (Levy et al., 2016, 2018; Pratt et al., 2016; Zebarjadi et al., 2021), was used in this study. The stimuli consisted of 108 digital color pictures of limbs (half) in physical pain such as injuries or wounds to depict painful (P) conditions (to elicit pain empathy) and (half) in nonpainful (N) conditions (to control for other factors). Stimuli were displayed randomly in a standardized size at the center of the monitor with a visual angle of 20.96° × 15.37° for 1 s, with an interstimulus interval of 2.5 to 3.3 s. In addition, we randomly presented twirl filler trials using a short twisted movement in new stimuli to maintain participants’ attention, and participants were trained to press a response button when they detected these twirl stimuli. The filler trials were not included in the data analysis.

#### Procedure

The experiment was programmed by E-Prime software (Psychology Software Tools). Participants were placed in a supine position inside the MEG system, facing the screen projecting the stimuli, and were instructed to maintain a relaxed posture, avoid moving their limbs, and focus their attention on the presented stimuli. The participants underwent MEG screening using a whole-head 248-channel magnetometer array (4-D Neuroimaging, Magnes 3600 WH) inside a magnetically shielded room while observing the stimuli on the screen. To track the participants’ head positions relative to the sensor, five coils were attached to their scalps. To minimize environmental noise, reference coils were placed ∼30 cm above the participants’ heads and aligned with the *x*-, *y*-, and *z*-axes. The sampling rate was set at 1,017 Hz, and a bandpass filter limited the frequency range to 1–400 Hz. After the scanning session, participants filled out several questionnaires to evaluate their empathic level and ideology.

#### Brain data analysis

##### Sensor analysis

The analysis for this study was done by MATLAB (MathWorks) and the FieldTrip software (Oostenveld et al., 2011). The data were bandpass filtered with the frequency range of 1–40 Hz, and eye and heart artifacts were eliminated from the raw data, using independent component analysis (ICA). The runICA algorithm was used with the rejection threshold of 4 × 10^−12^. Components corresponding to artifacts were identified through visual inspection of their time courses and topographies, focusing on patterns characteristic of eyeblinks and heartbeats. In addition, we visually inspected the data and discarded any remaining bad trials. Data were analyzed in alignment with the onset of the stimuli detected by the trigger. Based on previous MEG studies on empathy (Whitmarsh et al., 2011; Levy et al., 2018; Zebarjadi et al., 2021), epochs of 2 s after each stimulus onset were selected for further examination. To calculate the fast Fourier transform (FFT) on short sliding time windows of 500 ms, Hanning tapers were applied to each sensor data, and time–frequency representations (TFRs) of power for the alpha range (6–14 Hz) and each trial were calculated. Subsequently, the power estimates were averaged across all the trials and the statistical contrast between the two conditions (pain vs neutral) was calculated and adjusted for multiple comparisons, using a nonparametric permutation approach (more detail was provided below, Statistical analysis). Finally, by averaging over the significant time–frequency (TF) window on the peak sensor, a single averaged power value for each subject was obtained and the contrast between the averaged power values of subjects in the two opposing political groups was calculated.

##### Source analysis

To localize the source, we digitized the head shape of the participant during the MEG measurement (Polhemus FASTRAK digitizer) and used it to modify an MNI template using SPM8 (Wellcome Centre for Imaging Neuroscience, University College London, www.fil.ion.ucl.ac.uk) to build a single shell brain model for each subject. We divided the brain volume of the subject into 1 cm grids using a linear transformation. This individualized grid and the statistically significant TF window, detected through sensor-level analyses, were used as basic information for implementing beamforming techniques. For each grid position, spatial filters were created to selectively pass activity in the defined time window and from the specific location of interest while suppressing irrelevant activity. In the next step, we extracted the peak activity coordinates and conducted a virtual channel (VC) analysis on them to examine the alpha power changes for each experimental condition over time. Similar to the sensor-level analysis, we calculated a single averaged power value for each subject by averaging over the significant TF window on the peak source. Eventually, the contrast between the averaged power values of subjects in the two opposing political groups was calculated.

##### Statistical analysis

As typically implemented in MEG studies of empathy (Whitmarsh et al., 2011; Levy et al., 2016, 2018; Zebarjadi et al., 2021, 2023), we extracted a neural representation of empathy and then applied an independent *t* test to compare the representations in the two tested groups (i.e., rightists vs leftists). Power representations of empathy processing were computed for each subject, channel, frequency, and time. These *t* values were pooled over all group participants to define the test statistic. The TF clusters with significant effects at the random effects level were searched. The results were corrected for multiple comparisons along the time, frequency, and channel dimensions. To evaluate the multiple-comparisons corrected significance thresholds for a two-sided test, the smaller of the two fractions was then retrained and divided by 1,000. The proportion of values in the randomization distribution that exceeded the test statistic represents the Monte Carlo significance probability, known as the *p* value (Maris and Oostenveld 2007). This cluster-based nonparametric approach allowed to correct for multiple comparisons in all brain analyses. For nonsignificant results, Bayesian factors were computed in order to evaluate whether these results are supportive of the null hypotheses.

### Study 2

#### Participant

Forty-eight healthy native Finnish participants (37 females; mean age ± SD, 19.3 ± 1.71, 22 politically rightist and 26 politically leftist) were recruited on the Aalto University campus for this study. Subjects underwent screening for MEG compatibility and their medical background regarding psychiatric and neurological conditions before recruitment. In addition, participants were asked to report their demographics (such as gender and age) and political ideology. One subject was excluded after data acquisition measurement due to huge noises caused by the dental wire. Instructions were provided in the participants’ native language (Finnish), and they received compensation for taking part in the study. The study was approved by the ethics committee at Aalto University, and the consent form was signed by all participants.

#### Stimuli

The stimuli employed in this study were identical to those used in Study 1. Every three stimuli of the same type were randomly grouped into a block, and blocks were randomly presented to the participants. Each stimulus was displayed in a standardized size at the center of the monitor with a visual angle of 20.96° × 15.37° for 1 s with 3–3.5 s interstimulus intervals and ∼15 s interblock intervals. Similar to Study 1, the same techniques to maintain participants’ attention were employed.

#### Procedure

The experiment was programmed by Presentation software (Presentation; Neurobehavioral Systems). The participant sat in a relaxed position inside the MEG scanner, in front of a screen presenting the stimuli, and was asked to avoid movement during the measurement. Participants’ brain activity was captured using a whole head 306-channel MEG (Elekta Neuromag), located at the MEG Core section of Aalto neuroimaging infrastructure at Aalto University. The MEG device was in a magnetically shielded room that was equipped with an active noise cancellation system and a three-layer covering to minimize external magnetic fields. To ensure accurate measurements, five head position indicator (HPI) coils were attached to the participants’ scalps. The positions of the coils were recorded for each individual, and continuous HPI was applied throughout the recording. Additionally, electrooculography (EOG) electrodes were used to record eyeblinks and saccades during the measurement. The sampling rate was 1,000 Hz and a bandpass filter (0.1–330 Hz) was applied. After the MEG measurement, participants filled out several questionnaires to evaluate their empathic level and ideology.

#### Brain data analysis

In this study, the preprocessing was done using the MNE-Python toolbox (Gramfort et al., 2013). MaxFilter software (Elekta Neuromag) was used to filter the raw MEG data, reducing measurement artifacts and magnetic interference, and compensating for head movements. Then the data were bandpass filtered at 1–40 Hz, and eye and heart artifacts were detected and removed by the ICA method. The fastICA algorithm was used with the rejection threshold of 4 × 10^−12^ and 4,000 × 10^−13^ for mag and grad, respectively. Artifact-related components were identified by visually inspecting their time courses and topographies, particularly to typical patterns of eyeblinks and heartbeats. In the next step, similar to previous empathy studies looking into induced oscillatory responses, the epoch of 2 s after the stimuli onset was selected. Sensor, source, and statistical analysis for Study 2 were done almost in the same way as done in Study 1, using MATLAB (MathWorks) and the FieldTrip software to compare the findings of the two studies. The only difference was that the single shell brain model that was built based on the individual MRI and not the digitized head shape during MEG.

#### Self-reported measures

##### Political ideology scale

Participants rated their general political ideology using a 7-point political ideology scale ranging from 1 (extreme rightist) to 7 (extreme leftist). Participants who rated 1–3 on the self-reported political ideology scale explicitly reported that they are rightists (extreme, medium, light), and we classified them as the rightist group, and those who rated 5–7 explicitly reported that they are leftists (light, medium, extreme), and we classified them as the leftist group. For the Israeli dataset, we assessed the subjects’ reading habits by asking them to rate their preferences for two national newspapers in Israel: the left-leaning Ha’aretz and the right-leaning Israel Hayom on a scale from 1 (not at all) to 7 (all the time). These reading habit ratings were used first to verify the results of the political ideology scale and second to assign those who scored 4 to either the leftist or rightist group. No inconsistency was found between the results of the political ideology and reading habit scales. For the Finnish dataset, the validation of the political ideology scale and categorization of those who scored 4 were done based on other scales such as the initial online survey and the right-wing authoritarian (RWA) scale.

##### RWA scale

Participants provided ratings on items related to authoritarianism on a scale ranging from 1 (strongly object) to 7 (strongly agree). These items were derived from questionnaires on right-wing authoritarian (RWA; Altemeyer, 1983). The purpose of this scale was to assess the extent to which participants showed respect for and supported traditional values promoted by authorities. Previous research has indicated that individuals who scored higher on this scale typically exhibited a greater inclination toward right-wing political ideology (Manganelli Rattazzi et al., 2007).

##### Vicarious Pain Questionnaire

Participants were asked to watch 16 painful videos and answer questions related to perceived pain while watching each video. This scale measures pain perception (Grice-Jackson et al., 2017) and evaluates vicarious pain (i.e., pain empathy) from a more ecologically valid perspective. The two main questions used in the current study measure pain intensity and discomfort experienced by watching each stimulus, respectively. In total, 14 out of 124 participants did not complete the Vicarious Pain Questionnaire (VPQ) ratings.

##### Interpersonal Reactivity Index

Participants rated “empathic concern” (EC) and “perspective taking” (PT) subscales of the Interpersonal Reactivity Index (IRI) questionnaire (Davis, 1983) to assess their trait empathy.

## Results

### Whole sample

**Figure 1.**
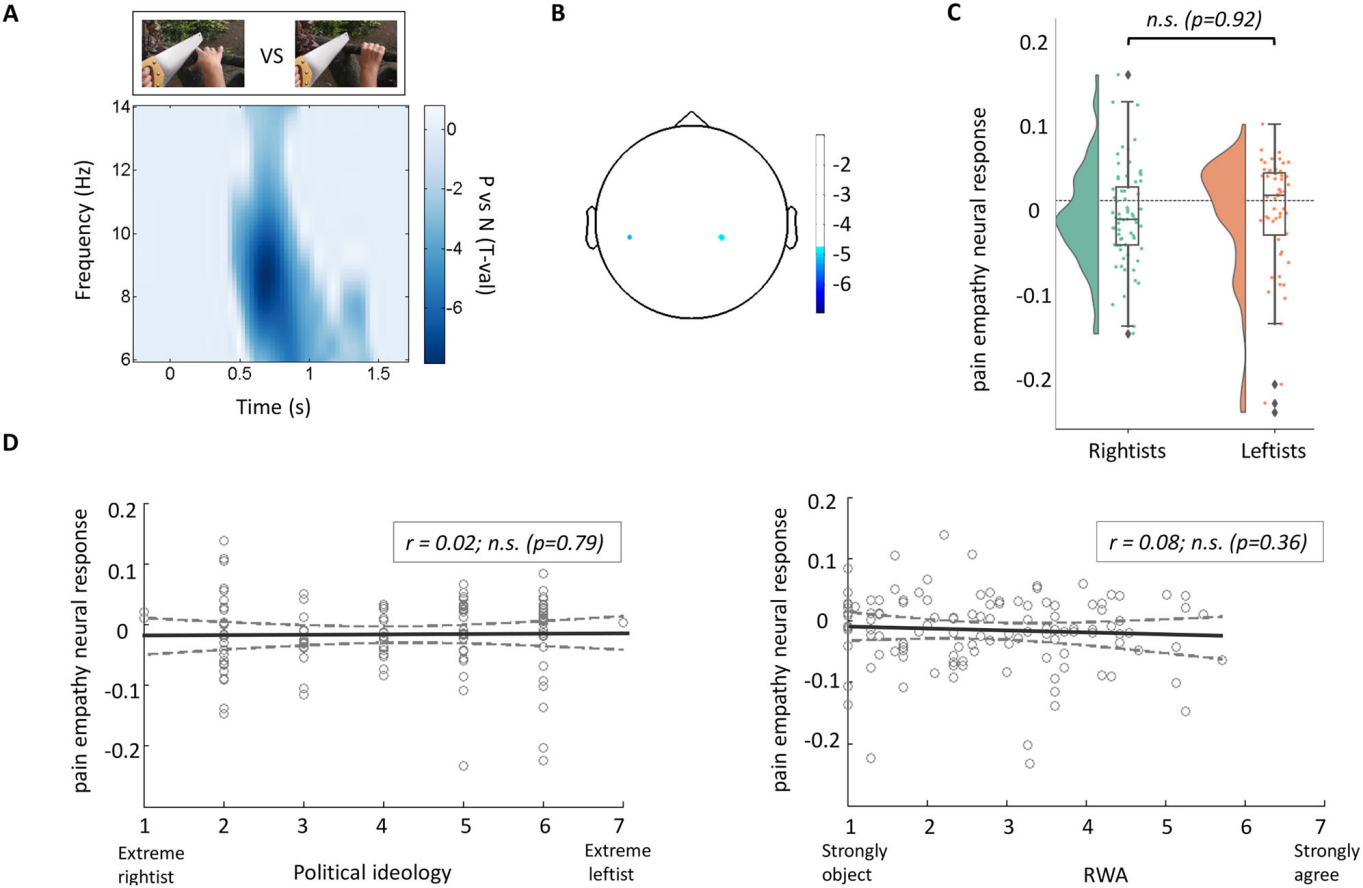
Pain empathy—the neural response. ***A***, TFR (bottom panel) of the pain empathy contrast pain versus no-pain (see top left image for stimulus example). ***B***, Topographic MEG sensor representation of the same pain empathy contrasts each dataset (compare SI). ***C***, Raincloud histograms of pain empathy (extracted from TFR representation) per each political group *D*, Parametric representation of pain empathy as a function of political ideology self-reports (left panel) and right-wing authoritarianism ratings (right panel).

The sensor analysis of all 124 subjects, contrasting painful and nonpainful conditions, determined a significant alpha power suppression pattern between the two conditions (negative *p*_cluster-cor_ < 0.001; *T* = −7.74; permutation test) with greater desynchronization in the pain condition, in the time window of 500–900 ms. The number of frequencies, time points, and channels for this permutation test were 9, 41, and 1, respectively. Figure 1, *A* and *B*, represents the TF representation on all subjects and the topographic MEG sensor representation of the same pain empathy contrast of each dataset, respectively. To evaluate the difference between the two political groups (68 rightist and 56 leftist subjects), a raincloud histogram is illustrated in Figure 1*C*, representing the power contrast values between the two conditions in the significant TF window (*F* = 6–14 Hz; *T* = 500–900 ms) for subjects in each political group. In contrast to earlier findings in (Zebarjadi et al., 2023), the difference between the leftists’ and rightists’ empathy levels is not significant (*p* = 0.92, independent *T* test). Bayesian factor showed moderate support for the null hypothesis (BF = 0.19), namely, that there is no difference between rightists and leftists. In addition, nonsignificant correlations between sensor neural results, and two different political-related scales (political ideology rating and RWA rating), are represented in Figure 1*D*.

Due to the use of different MEG machines with varying numbers of sensors for each dataset of the current study, a source analysis was conducted separately for each study. This enabled us to evaluate the empathy difference to vicarious physical pain both at the sensor and source levels between the rightist and leftist groups.

#### Study 1

**Figure 2.**
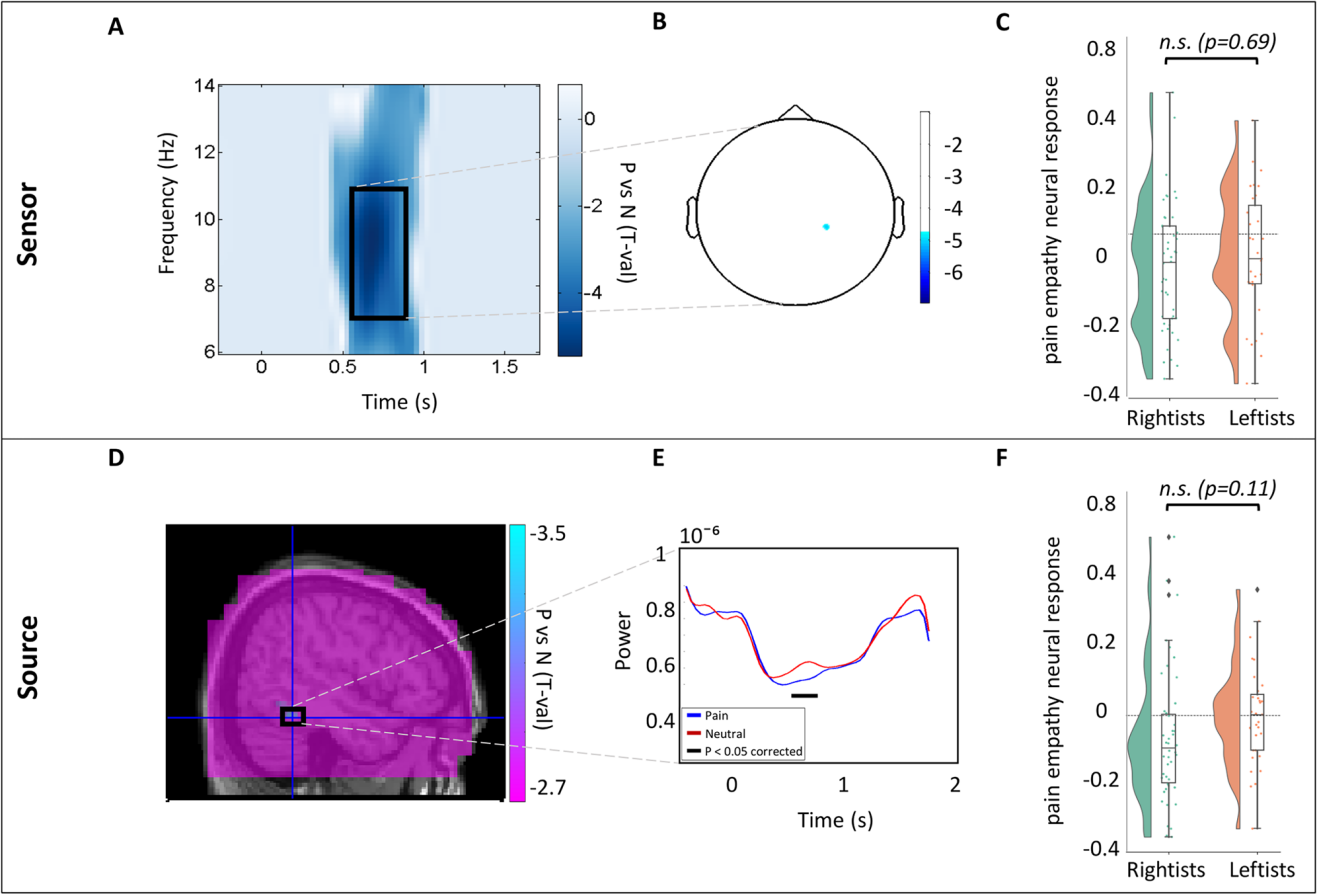
***A***, TFR of statistical contrast between the two conditions on 77 subjects. ***B***, Topographic representation of the most suppressed sensor. ***C***, Raincloud histogram, indicating alpha power change ratio in each political group, calculated on the peak sensor and averaged over the significant TF window. ***D***, Sagittal view of alpha suppression peak source in the brain, detected by beamforming technique. ***E***, Alpha power temporal changes on peak source in both conditions. ***F***, Raincloud histogram, indicating VC power ratio in each political group, averaged over the significant TF window.

Whole-brain sensor analysis (contrasting the two conditions) on 77 participants within the alpha range, shown in Figure 2*A*, revealed a significant suppression in alpha power (negative *p*_cluster-cor_ < 0.001; *T* = −5.4; permutation test). The number of frequencies, time points, and channels for this permutation test were 9, 41, and 1, respectively. This significant alpha suppression at the sensor level during the physical pain empathy task may imply a possible pain empathy effect in this population. We selected this TF window (*F* = 7–11 Hz; *T* = 600–850 ms) based on the power statistics and conducted further analysis on this window. The black rectangle in Figure 2*A* represents the selected TF window that has been used for further analysis. The illustrated peak sensor in Figure 2*B* was detected by averaging over the selected TF window. To evaluate the difference between the groups, we first examined this contrast at the sensor level. As shown in Figure 2*C*, the contrast of alpha power changes in the detected peak coordinate between the two groups was not significant (*p* = 0.69; independent *T* test), and Bayes factor (BF = 0.25) showed moderate support for the null hypothesis. To examine the source of alpha suppression in the brain, we conducted beamforming analysis on the selected TF window with the peak frequency at 9 Hz and 5% regularization. Figure 2*D* represents the significant source found at the right inferior temporal cortex (negative *p*_cluster-cor_ = 0.004; *T* = −3.5; permutation test). The number of frequencies, time points, and channels for this permutation test were 5, 6, and 1, respectively. To evaluate the alpha power temporal changes in response to the physical pain and neutral conditions, we conducted virtual channel ROI analysis on the peak coordinate extracted from the beamforming analysis on peak frequency (9 Hz) and over the whole time range. As illustrated in Figure 2*E*, a significantly greater suppression in response to physical pain compared with neutral stimuli was found (negative *p*_cluster-cor_ = 0.012; *T* = −3.1; permutation test). The virtual channel analysis enabled the evaluation of contrast in alpha power changes over time at the source level between the two political groups. Similar to the sensor level, the difference between the alpha power changes between rightists and leftists, shown in Figure 2*F*, was statistically nonsignificant (*p* = 0.11; independent *T* test), and Bayesian analysis showed anecdotal support in null hypothesis (BF = 0.75).

#### Study 2

**Figure 3.**
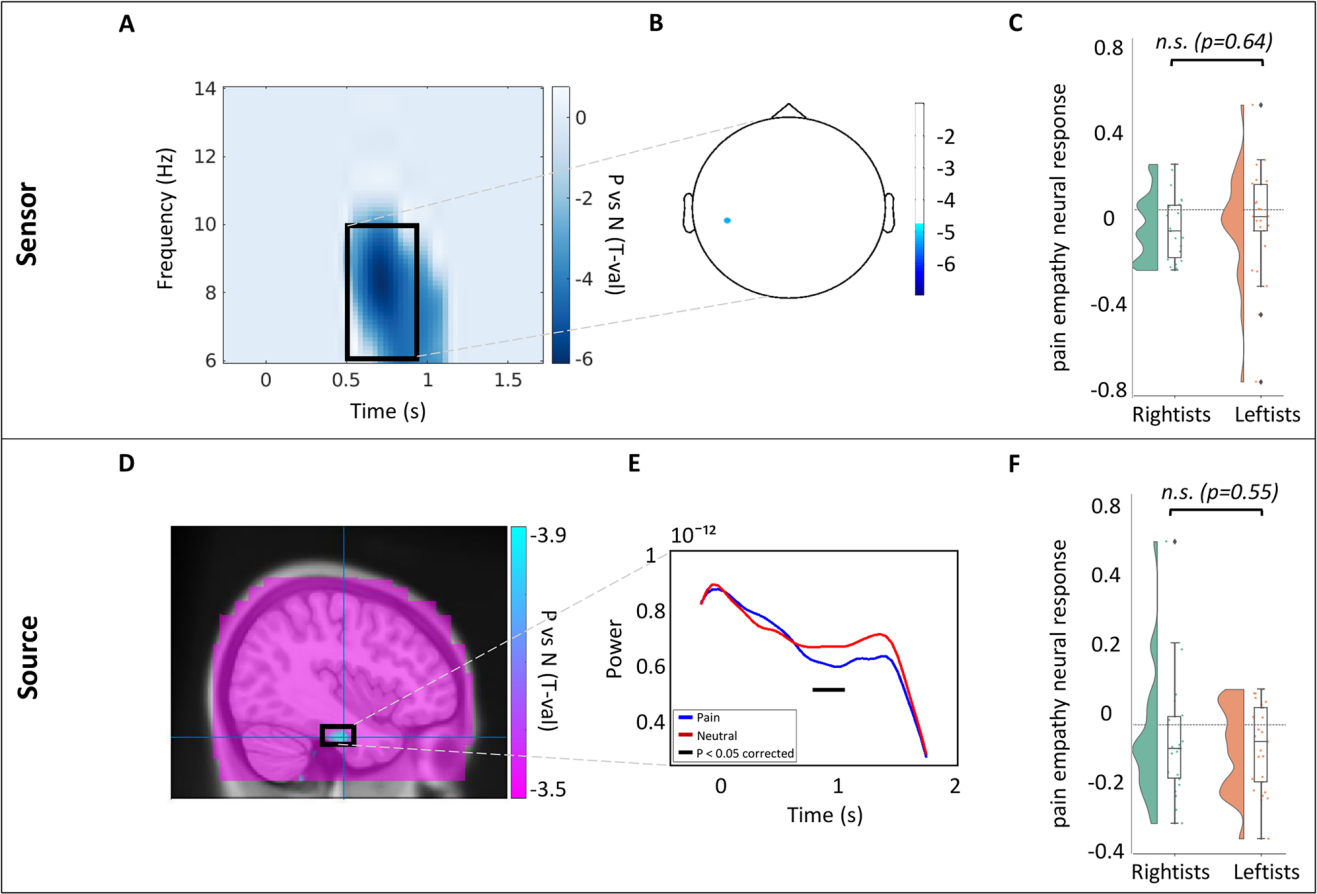
TFR of statistical contrast between the two conditions on 47 subjects. ***B***, Topographic representation of the most suppressed sensor. ***C***, Raincloud histogram, indicating alpha power change ratio in each political group, calculated on the peak sensor and averaged over the significant TF window. ***D***, Sagittal view of alpha suppression peak source in the brain, detected by beamforming technique. ***E***, Alpha power temporal changes on peak source in both conditions. ***F***, Raincloud histogram, indicating VC power ratio in each political group, averaged over the significant TF window.

The whole-brain sensor analysis (contrasting the two conditions) on 47 subjects within the alpha range, depicted in Figure 3*A*, represents a significant decrease in alpha power (negative *p*_cluster-cor_ = 0.006; *T* = −5.9; permutation test). The number of frequencies, time points, and channels for this permutation test were 9, 41, and 1, respectively. This significant alpha suppression may indicate an effect in this population in response to pain empathy stimuli. To examine the source of the suppression pattern, we first checked the source on the peak TF window based on the power statistics (*F* = 7–9 Hz; peak frequency = 8 Hz; *T* = 650–900 ms) and with 5% regularization, but no significant source was identified within this specific TF window (negative *p*_cluster-cor _= 1; *T* = −3.7; permutation test). However, looking at the overall 124 subjects’ time range and wider frequency range (*F* = 6–10 Hz; peak frequency = 8 Hz; *T* = 500–900 ms) and without regularization provided a close to significant result (negative *p*_cluster-cor_ = 0.08; *T* = −4.85; permutation test), which should be considered with caution. The number of frequencies, time points, and channels for this permutation test were 5, 9, and 1, respectively. The black rectangle in Figure 3*A* shows the latter TF window that has been used for further analysis. Figure 3*B* indicates the peak sensor over the averaged TF window, and Figure 3*C* represents the power contrast values between the two conditions in this peak sensor for subjects in each political group with no significant difference (*p* = 0.64; independent *T* test), and Bayesian factor (BF = 0.32) showed moderate support for the null hypothesis, claiming there is no political differences. As illustrated in Figure 3*D*, the alpha suppression emanates from the source at the left inferior temporal region. Further virtual channel ROI analysis on the peak source coordinate at the peak frequency (8 Hz) and over the whole time range (Fig. 3*E*) indicates a significantly greater suppression in the physical pain versus neutral conditions (negative *p*_cluster-cor_ = 0.012; *T* = −2.65; permutation test). Similar to the sensor-level results, Figure 3*F* represents the statistically nonsignificant difference in alpha power changes between individuals in two opposing political groups (*p* = 0.55; independent *T* test). The Bayesian factor of this nonsignificant effect is a mild–moderate evidence supporting the null hypothesis (BF = 0.34).

After conducting the source analysis in each dataset separately, we used the single value extracted for each subject and evaluated the correlation between the source power values for all 124 subjects and both political-related ratings. The correlation between the source level results and both political ideology rating (*r* = 0.04; *p* = 0.66) and RWA rating (*r* = 0.003; *p* = 0.97) were statistically nonsignificant.

### Between political groups analysis

We further examined the contrast between the political groups over the whole TF range (*F* = 1–40 Hz; *T* = 0–2 s) once for all 124 subjects together at the sensor level and then for the data in each study separately, at both sensor and source levels. The comparisons at the sensor level were done over all sensors and the whole time range and showed nonsignificant differences for all subjects (negative *p*_cluster-cor_ = 0.7; positive *p*_cluster-cor_ = 0.5; permutation test), for Study 1 (negative *p*_cluster-cor_ = 0.4; positive *p*_cluster-cor_ = 0.9; permutation test) and Study 2 (negative *p*_cluster-cor_ = 0.6; positive *p*_cluster-cor_ = 0.2; permutation test). Besides, for each dataset, we averaged over the small TF window to check whether any significant sensors are different between groups but we have not found any sensor neither in Study 1 nor Study 2. Similarly, the contrast between the two political groups at the source level was statistically nonsignificant for Study 1 (negative *p*_cluster-cor_ = 0.1; positive *p*_cluster-cor_ = 1; permutation test) and for Study 2 (negative *p*_cluster-cor_ = 1; positive *p*_cluster-cor_ = 0.1; permutation test).

### Behavioral analysis

As explained in the Materials and Methods section, VPQ and IRI scales evaluate the perception of vicarious pain and trait empathy, respectively. In the VPQ scale, one measure evaluates the intensity of pain (VPQ 1), and the other measure examines the associated discomfort while watching the stimuli (VPQ 2). In the IRI scale, the evaluation was done for EC and PT measures. The statistical details for these measures in each study are provided in Table 1.

**Table 1.** Statistical details for each self-reported measure in each dataset

	Study 1				Study 2			
	VPQ 1	VPQ 2	IRI (EC)	IRI (PT)	VPQ 1	VPQ 2	IRI (EC)	IRI (PT)
Cronbach’s *α*	0.94	0.89	0.75	0.78	0.97	0.91	0.78	0.72
Mean	2.20	4.09	5.83	5.70	2.50	5.44	5.74	5.44
Standard deviation	2.21	1.90	0.90	0.99	2.58	2.27	0.87	0.75

In the next step, the interplay between these measures and neural findings (behavioral–neural analysis) and their contrast among political groups (behavioral–political analysis) were evaluated.

#### Behavioral–neural analysis

We correlated each of these self-reported findings with neural results (the contrast between the conditions) at both sensor and source levels. For the sensor-level neural results, the correlation was only significant with one measure, the neural(sensor)-VPQ 2 (*r* = −0.22; *p*_FDR-cor_ = 0.02) and not with the other measures as follows: neural(sensor)-VPQ 1 subscale (*r* = −0.13; *p* = 0.16), neural(sensor)-IRI EC (*r* = 0.02; *p* = 0.82) and neural(sensor)-IRI PT (*r* = −0.05; *p* = 0.57). For the source-level neural results, no significant correlation was found between them and self-reported finding neural(source)-VPQ 1 (*r* = −0.12; *p* = 0.22), neural(source)-VPQ 2 (*r* = −0.14; *p* = 0.14), neural(source)-EC (*r* = −0.03; *p* = 0.72) or neural(source)-PT (*r* = −0.01; *p* = 0.9) subscales. These results may indicate that neural measure is more related to behavioral real-life measures of sensitivity to vicarious pain (measured by VPQ) as a correlation detected for neural-V, rather than self-reported trait empathy (measured by IRI). In addition, for the Finnish dataset, we evaluated the correlation between alpha suppression at the peak coordinate and the self-reported ratings of pain stimuli and the correlation was significant (*r* = −0.3; *p* = 0.44).

#### Behavioral–political analysis

**Figure 4.**
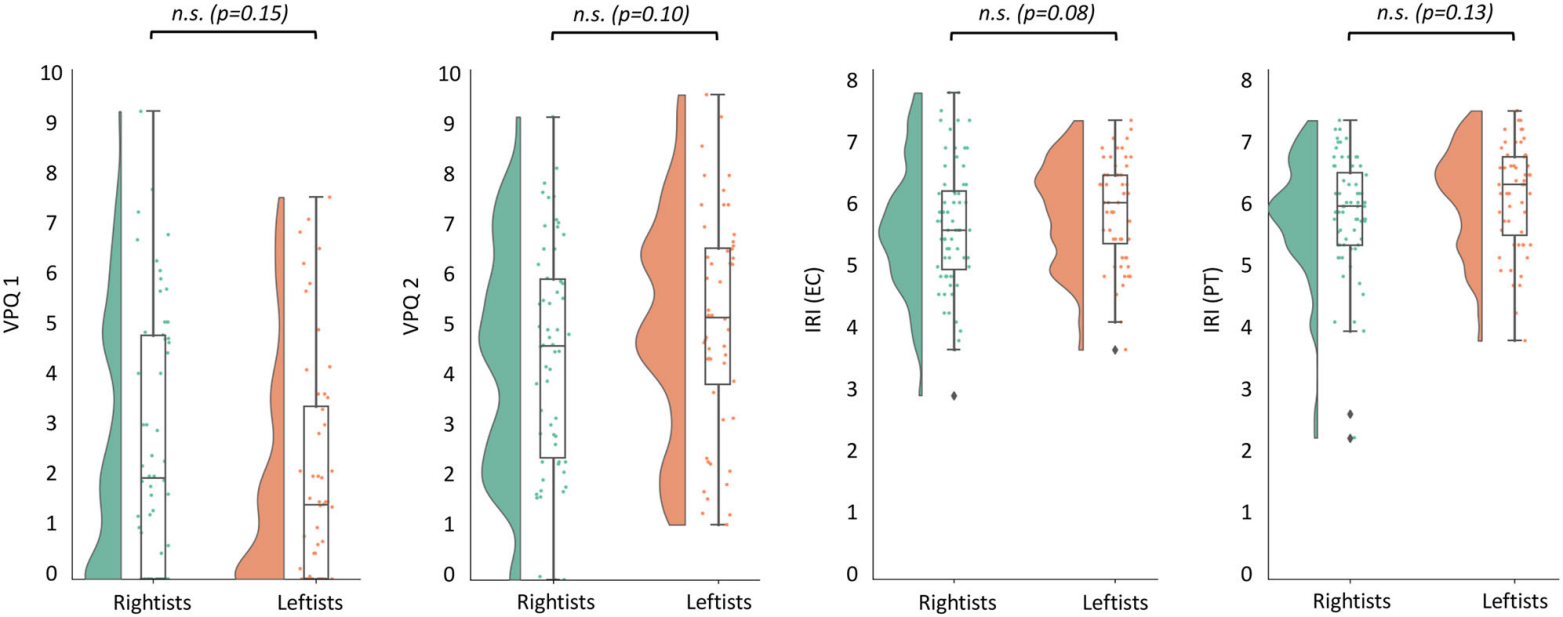
Pain empathy—the behavioral and self-reported measures. This indicates the contrast of behavioral (VPQ) and self-reports (IRI) measures of empathy in the two opposing political groups, all provided nonsignificant contrast V: *p* = 0.15, mean = 2.33, SEM = 0.21; V: *p* = 0.10, mean = 4.66, SEM = 0.19; IRI(EC): *p* = 0.08, mean = 5.8, SEM = 0.08 and IRI(PT): *p* = 0.13, mean = 5.6, SEM = 0.08.

To examine the behavioral contrast between the two opposing political groups, we conducted an independent *t* test between political groups on the VPQ and IRI subscales for all 124 subjects. Figure 4 represents the contrast between the political groups for each VPQ and IRI subscales. Similar to the neural results, none of the VPQ (VPQ1: *p* = 0.15, mean = 2.33, SEM = 0.21; VPQ2: *p* = 0.10, mean = 4.66, SEM = 0.19) and IRI [IRI(EC): *p* = 0.08, mean = 5.8, SEM = 0.08; (IRI(PT): *p* = 0.13, mean = 5.6, SEM = 0.08] contrasts were statistically significant. In addition, for the Finnish dataset, we calculated the contrast of self-reported ratings of pain stimuli between the two political groups and it was not significant (*p* = 0.22; mean = 2.12; SEM = 0.136).

Additionally, the correlation between political-related ratings and behavioral empathy measures was evaluated. The correlation between the political ideology rating and all four behavioral measures was statistically nonsignificant: VPQ 1 (*r* = −0.17; *p* = 0.08), VPQ 2 (*r* = 0.12; *p* = 0.21), EC (*r* = 0.16; *p* = 0.07), and PT (*r* = 0.05; *p* = 0.58). Similarly, the correlation between the RWA rating and VPQ 1 (*r* = −0.01; *p* = 0.94), EC (*r* = −0.01; *p* = 0.95), and PT (*r* = 0.01; *p* = 0.89) were statistically nonsignificant. For VPQ 2, there was a weak negative correlation (*r* = −0.21; *p*_uncorrected_ = 0.03), but it did not survive FDR correction. These correlations are consistent with what is found in Figure 4 and indicate that the behavioral measures are not affected by political affiliation and RWA.

## Discussion

The current study set out with the aim of assessing the association of individuals’ political ideology and psychological traits, particularly empathy toward vicarious physical pain, using both neuroimaging and self-reported measures in a diverse sample. The past two decades have seen the emergence of a lively debate about ideological asymmetries in psychological processes (Brandt et al., 2014a; Jost, 2017). Our study contributes a new understanding to this debate, from the outlook of two relatively separate sets of literature.

First, in that broad domain of research, what has drawn particular interest was the investigation of whether political ideologies can be explained by biopsychological traits; to investigate this, a series of studies have mainly centered on the neural and physiological responses to images depicting vicarious pain, disgust or the sensation of threat (Thorisdottir et al., 2007; Oxley et al., 2008; Ahn et al., 2014; Brandt et al., 2014b; Bakker et al., 2020; Smith and Warren, 2020; Jost, 2021). The pioneering study in this line was conducted by Oxley et al. (2008), who discovered a potential association between political attitudes and physiological sensitivities to sudden sounds and threatening visual stimuli (Oxley et al., 2008). Their investigation suggested that individuals with lower sensitivity to such stimuli may exhibit a greater inclination toward endorsing liberal immigration policies and conversely, those with higher physiological reactivity to these stimuli display a tendency for increased defense expenditure and expressions of patriotism (Oxley et al., 2008). Similarly, Ahn et al. employed neuroimaging and machine-learning techniques to examine this association and posited that neural responses to emotionally evocative nonpolitical stimuli (i.e., disgusting stimuli) can be highly predictive of political orientation (Ahn et al., 2014). However, in 2020, Bekker and colleagues failed to replicate the results of the Oxley et al. study and did not find any evidence to support the claim that conservatives have stronger physiological responses to threats than liberals (Bakker et al., 2020). Likewise, Brandt et al. challenged the assumption that liberals and conservatives are fundamentally different in their psychological responses to threatening stimuli and proposed an alternative perspective, indicating both ideological groups react in similar ways to such stimuli (Brandt et al., 2014b). In addition, in a recent comprehensive review, Smith and colleagues assessed the empirical records concerning the connection between political beliefs and individual differences in sympathetic nervous system (SNS) activity in response to disgusting and threatening stimuli and found mixed empirical evidence (Smith and Warren, 2020). Overall, the interplay between political inclination and individual psychophysiological traits remains inconclusive, and consequently, the generalizability of the findings comes into question, prompting the need for further empirical exploration and using a more accurate and objective measurement approach to understand this association, as suggested by Smith and Warren.

In the current study, we examined this association by collecting both neuroimaging and behavioral data in two distinct countries. Both datasets in this study utilized stimuli portraying physical pain experienced in the protagonist’s body part, enabling shared affective and sensory responses in the observer. The investigation of vicarious physical pain shares similarities with the investigation of disgust and threat (relying on the International Affective Picture System) which was used in some of the studies cited above (Oxley et al., 2008; Ahn et al., 2014; Bakker et al., 2020; Smith and Warren, 2020). The response to observing the stimuli was examined at the sensor level for both datasets together and separately and at the sensor and source levels for each dataset. The neural result was evaluated by inspecting alpha power changes in the time window of 0–2 s after stimulus onset. Previous EEG/MEG studies on pain empathy have consistently demonstrated alpha power suppression within a few hundred milliseconds after stimulus onset (Perry et al., 2010; Whitmarsh et al., 2011; Motoyama et al., 2017; Levy et al., 2018; Zebarjadi et al., 2021; Zebarjadi and Levy, 2023). For example, these studies observed significant alpha suppression in the sensory cortices when participants viewed pain-related images compared with nonpain images. They proposed that, according to the “gating to inhibition” hypothesis, alpha power suppression plays a disinhibitory role in brain regions involved in empathetic responses. The source of the alpha suppression pattern reflecting physical pain empathy was detected in the inferior temporal region, previously shown to be activated while perceiving human body parts and making self/other distinctions (Hooker et al., 2010). Occipitotemporal involvement in pain empathy has also been identified in a previous meta-analysis of fMRI studies (Fallon et al., 2020). However, it is important to note that MEG has limitations in source localization, as it relies on the beamforming technique to identify the source of brain activity. Furthermore, to our knowledge, there is no meta-analysis of MEG studies on pain empathy, but rather of fMRI studies. These factors may explain why the detected source appears slightly more anterior than reported in the aforementioned meta-analysis. Nonetheless, the consistent detection of this source across both Study 1 and Study 2, using different MEG systems and populations, provides strong evidence that the observed contrast taps the empathy process. By splitting the subjects into two opposing political groups, we detected no asymmetry in pain empathic response at the sensor or source level. Additionally, Bayesian factors calculated and supported the null hypotheses in an anecdotal to moderate level. Consistency of the findings—nonsignificant effect and BF < 1 across all analyses—further support the null hypothesis claim that there is no effect in this task between the two groups. This finding provides new insights into the role of political ideology in the empathy level of individuals and contributes to ongoing debates on the association between political ideology and psychological traits. It is in agreement with the studies indicating ideological symmetry in psychological responses to threatening or disgusting stimuli (Brandt et al., 2014b; Bakker et al., 2020) and supports an earlier study by Brandt et al. suggesting a complex but solvable relationship between political ideology and psychological traits (Brandt and Bakker, 2022).

The second set of literature comes from the fields of political and social psychology. Recent literature has presented contradictory findings about the link between empathy and political ideology (Wagaman and Segal, 2014; Waytz et al., 2016; Hasson et al., 2018; Morris, 2020; Casey et al., 2023; Zebarjadi et al., 2023). A number of these investigations suggested a heightened empathy level within the leftist group in comparison with the rightist group (Wagaman and Segal, 2014; Hasson et al., 2018; Morris, 2020), while others failed to detect similar outcome (Waytz et al., 2016; Casey et al., 2023). However, the majority of these studies relied on self-reported questionnaires to examine the empathy level, with only one recent study employing an objective measure, namely, neuroimaging, to assess the level of empathy (Zebarjadi et al., 2023). Similar to the latter study, we employed neuroimaging, i.e., MEG, to evaluate empathy level in the current study. Howbeit, the finding in the present research is contrary to earlier research suggesting a neural asymmetry in empathic response to vicarious emotional suffering among leftists and rightists (Zebarjadi et al., 2023). A possible explanation for this discrepancy might be that in the earlier study (Zebarjadi et al., 2023), participants were exposed to a paradigm that was based on stories and pictures of people in emotional situations and instructed to mentalize with the stories. The neural marker of empathy to vicarious emotional suffering was identified in the TPJ region, a brain area consistently associated with Theory of Mind and perspective-taking (Lamm et al., 2011). Given that mentalizing ability or tendency might vary among individuals with different ideological foundations and values stronger TPJ activation in the leftist group compared with the rightist group may be linked to differences in their perspective on the social world. In contrast, the stimuli in the current study depict pain in body parts, which is less influenced by the observer’s ideological values. Besides, it might induce an internal threat rather than a collective threat that may possibly be relevant to right-wing attitudes (Onraet et al., 2013). The difference in the neural mechanism underlying various types of empathy is in agreement with the prior findings indicating distinct brain networks involved in emotional empathy (linking to mentalizing or sadness), versus physical pain empathy (linking to sensations of disgust or perceiving threat; Lamm et al., 2011; Bruneau et al., 2012). This and other studies indicated that the engagement of various empathy components may vary depending on the stimuli eliciting empathic responses (Lamm et al., 2011; Bruneau et al., 2012). For instance, the meta-analysis by Lamm et al. (2011) highlighted distinct brain regions activated during empathy depending on the type of experimental paradigm. They discussed that images of body parts in painful situations activate neural structures involved in understanding and predicting the outcomes of these situations, thereby triggering inferences about their affective consequences. In contrast, stimuli associated with TOM elicit inferences about self- and other-related social information and facilitate the sharing of other’s state based on one’s own prior experiences and knowledge (Lamm et al., 2011). The current results suggest that the previously reported findings on the greater level of empathy in leftists compared with the rightists may apply to a distinct type of empathy and may not be generalized to other manifestations of empathy. In addition, contrary to previous self-reported studies (Wagaman and Segal, 2014; Hasson et al., 2018), the evaluation of self-reported data in the current research revealed no differences in trait and state empathy between the two political groups. Nonetheless, with a smaller sample size in this study compared with the previous self-reported studies, caution must be applied. It is also important to note that the detected null results in the current study might not necessarily indicate the absence of difference between the political groups, and future research with higher statistical power and sample variability may further explore this point. Furthermore, neural–behavioral analysis indicated a weak correlation between sensor neural results and one of the VPQ measures, whereas nonsignificant correlations were found between this neural finding and IRI measures. This may suggest that the neural measures are more closely associated with behavioral real-life sensitivity to vicarious pain rather than with self-reported trait empathy. However, no significant correlation was found between the source neural findings and behavioral or self-reported measures which might be due to the limitations imposed by the MEG measurement or lower sample size compared with the psychological studies, as reported in the previous research (Whitmarsh et al., 2011; DiGirolamo et al., 2019). In this study, self-reported vicarious pain ratings has not been measured for the Israeli dataset and are suggested to be incorporated in future research to provide further correlational analysis between the neural findings and self-reported pain response to the same stimuli. Moreover, a cross-national investigation by Malka et al. (2014) demonstrated a stronger link between personality traits and social conservatism in ideologically constrained nations (Malka et al., 2014). To account for cultural disparities, data for the current research was gathered in two distinct countries, Finland and Israel. The neural and behavioral outcomes demonstrated consistency across both populations, affirming the generalizability of the findings beyond a specific political context. Yet, it is noteworthy that the political dimension might slightly vary in different social contexts (e.g., liberal–conservative dimension is commonly used in the United States instead of the left–right dimension), and the results should be interpreted with caution. Despite the limitations, the combined evidence from neural and behavioral investigations, along with our recent study (Zebarjadi et al., 2023), addresses a point that was overlooked thus far (Wagaman and Segal, 2014; Waytz et al., 2016; Hasson et al., 2018; Morris, 2020; Casey et al., 2023): It provides a unique perspective on the interaction between empathy and political ideology by examining different subcomponents of empathy and the neural response that they trigger. It underscores the complexity of this association, emphasizing the importance of considering targeted components of empathy, societal contexts, and unbiased measurement approaches, such as neuroimaging techniques, when assessing empathic responses. Moreover, it enriches the ongoing debate regarding the relationship between political ideology and psychological traits (e.g., susceptibility to threat, disgust or pain) by further consolidating the recent view of ideological symmetry (Oxley et al., 2008; Ahn et al., 2014; Brandt et al., 2014b; Bakker et al., 2020; Smith and Warren, 2020) in vicarious pain perception. We would like to end with a note that sometimes null findings can be very informative and insightful for science.

## Synthesis

Reviewing Editor: Niko Busch, Westfalische Wilhelms-Universitat Munster Decisions are customarily a result of the Reviewing Editor and the peer reviewers coming together and discussing their recommendations until a consensus is reached. When revisions are invited, a fact-based synthesis statement explaining their decision and outlining what is needed to prepare a revision will be listed below. The following reviewer(s) agreed to reveal their identity: NONE. Note: If this manuscript was transferred from JNeurosci and a decision was made to accept the manuscript without peer review, a brief statement to this effect will instead be what is listed below.

# Synthesis

Your manuscript has been reviewed by an expert in Political Neuroscience and me. Together, we agree on the merit of your study, but also on the need to improve the manuscript. While reviewer #1 focuses on the overall rationale and interpretation in terms of political ideology and interpretation of empathy processing, my own comments below pertain mostly to the MEG data analysis. Moreover, we have made several comments regarding the statistical analysis. I encourage you to address each of these points carefully. Finally, I would like to ask you to consider making the data and analysis code publicly available.

## Comments on the MEG analysis and interpretation

- "depicting painful (P) conditions" -- Please give a brief description explaining how exactly pain is depicted on these images. Please specify how large the stimuli were in degrees of visual angle and how long they were presented.

- "artifacts were eliminated from the raw data, using independent component analysis" -- please specify ICA algorithm and the criteria that were used to select components for rejection.

- "onsets of the stimuli were detected" -- Does this refer to conventional triggers that were synchronized with image onsets? What exactly does "detection" refer to? Please clarify.

- Time-frequency analysis: please specify the exact frequency range of the "alpha band".

- "the statistical contrast between the two conditions (pain versus neutral) after adjusting for multiple comparisons was calculated" -- how was the test calculated? Which method was used for adjusting for multiple testing and how was the number of tests determined (number of frequencies, time points, channels, etc.)? How does this test relate to the cluster permutation test that is mentioned later on? If I understood correctly, the cluster test was computed in channel space (not source space), as well.

- "Initially, t-values, indicating the differences between the conditions, were computed for each subject, channel, frequency, and time... The results were corrected for multiple comparisons along the time and frequency dimensions" -- The correction should also account for the channel dimension.

- MEG data analysis is only reported for Study 1. Were Study 2 data processed in exactly the same way? Please clarify.

- "The sensor analysis of all 124 subjects determined a significant alpha power suppression pattern".

- The MEG results are often referred to as "alpha power suppression" or "a significant decrease in alpha power." If I understand correctly, this does not refer to changes in alpha power pre se (i.e. with respect to the pre-stimulus interval), but to differences between the two image categories. I think this should be stated explicitly, at least when such results are mentioned for the first time. Moreover, I think this would help explain the polarity of this effect. I suppose that the stimulus onset induces desynchronization under both conditions. Which condition desynchronizes more strongly when the t-value is negative?

- I think the rationale for focusing on this particular frequency band should be explained in more detail. How was this frequency band determined a priori to be the frequency range of interest? Moreover, the discussion of these results should be expanded. Specifically, how is alpha power thought to be related to empathy, and why? In the figures, the MEG effect is referred to as "pain empathy neural response," which seems to refer to a strong interpretation of what this signal reflects. What exactly is the evidence that alpha suppression is a correlate of *empathy* for the person depicted in these images rather than a correlate of something else, such as attention, interest, arousal, or physical characteristics of these images? How does the tentative role of the alpha rhythm for empathy jibe with the vast literature on the connection between alpha and arousal and attention?

# Reviewer 1

While previous research by the authors (Zebarjadi et al., 2023) showed that there is ideological asymmetry in cognitive-affective empathy, the authors now show that there is no evidence for ideological assymetry in empathy for pain. Even though I find this interesting, I am not entirely convinced about the author's rational of proposing a distinction in empathy type between this and their last study given that empathy for pain also involves affective, cognitive aspects of empathy and there is mentalisation in both cases (i.e., in response to distressing stories or seeing people in pain). I also question their justification of these being separate processes because "empathy for pain relies on bottom up processes".

I also ask the authors to provide more details about their "political ideaology measure". This seems to involve a 7 point likert scale but there is no mention of how where participants assigned to leftists vs rightists (e.g., median split?). Details about how/if it was validated, and whether it adequately captures the complexity of political ideology are also needed as examining differences between rightists and leftists is the main aim of the paper.

## Statistics:

1. The statistical analyses are not clearly explained in some sections and therefore I cannot judge if they are correct or not.

2. In some other parts it seems that the authors analysed the pain vs neutral conditions and then the leftist vs rightist on alpha supression. Given they have a 2 x 2 design ideally they should analyse them in a way to look for an interaction (e.g., 2 x 2 anova). It is also not clear if the ideological assymmetry was only analysed in the pain condition (or on both).

3. The authors seem to interpret the lack of significant differences in empathy responses between political groups as a straightforward null finding. However, it's crucial to consider that null results do not necessarily indicate the absence of an effect. Further analyses needed : for example Bayes factor to provide information about how weak or strong is the evidence in favour for the null or alternative hypothesis.

## Evaluation:

Note that I am not an expert on MEG so I cannot comment on the accuracy of the methods and analyses regarding this technique.

This is an interesting study into the potential lack of ideological asymmetry in empathy - at least in response to empathy for pain. While previous research by the authors (Zebarjadi et al., 2023) showed that there is ideological asymmetry in cognitive-affective empathy, the authors interpret their null significant results as pertaining to a subtype of empathy, namely empathy for pain. To investigate this matter, the authors used questionnaires and neuroimaging (MEG) methods. While I think this study has merit, I have some methodological and statistical concerns, as well as some suggestions. Note that I am not an expert on MEG so I cannot comment on the accuracy of the methods and analyses regarding this technique.

Please see my comments below:

Introduction

1. I am not entirely convinced about the author's rational proposing a distinction in empathy type between this and your last study (Zebarjadi et al., 2023) given that empathy for pain also involves affective, cognitive aspects of empathy and there is mentalisation in both cases (i.e., in response to distressing stories or seeing people in pain)

Methods

2. The method used to classify participants as politically left or right is briefly mentioned, involving a seven-point scale, and this needs to be better approached. For example, where participants assigned as leftists or rightists by doing a median split, or by only taking the extremes? The manuscript could also benefit from a more detailed description of this scale, e.g., how it was validated, and whether it adequately captures the complexity of political ideology. This is crucial as examining differences between these is the main aim of the paper.

Study 1 results

3. Overall, results need to be explained better. For example, explain what this means for non-technical audience: " Whole-brain sensor analysis on 77 participants within the alpha range, shown in Figure 2A, revealed a significant suppression in alpha power (negative Pcluster-cor < 0.001, T= -5.4, permutation test).

4. The authors report no changes in alpha suppression between rightists and leftists (t test), but a more nuanced approach would be a better fit to examine whether the results are specific for empathy for pain (relative to neutral) and differ by political ideology. This should include all conditions and interactions, e.g., a 2 (stimuli: neutral, painful stimuli ) x 2( ideology : rightist , leftist) anova. An ideal outcome would be a significant interaction effect, indicating that the empathetic response to pain (as reflected in alpha suppression) is not only specific to painful stimuli but also varies according to political ideology. Yet if non-parametric tests are necessary due to data distribution, then the authors could show this by, for example, looking at difference scores (although not ideal).

Study 2 Results

5. Results need to be written more clearly and explain what they suggest taken together.

6. If I understand correctly, there are two comparisons done separately at a source level : one comparing suppression between neutral and pain and another rightists between leftists. However, to investigate the research question, one would expect a difference between leftists and rightists in pain vs neutral conditions, and unless I am missing something, this isn't being tested here. See also point above in relation to study 1.

Behavioral analyses

7. Almost the whole entire first paragraph needs to be re-written (lines 317-326). For example, what do the VPQ subscales measure? This hasn't been explained earlier so we cannot follow. Or do the authors mean scale instead of subscale ? And the authors mention that they are correlating questionnaires to sensor-level results, but in response to what experimental condition? Also, Line 321 to 324 is not clear. And Line 324, 325, 326 needs to be better explained on how you got to that conclusion.

8. Did the authors collect vicarious pain ratings as a behavioural measure of pain in response to each picture? Correlational analyses between these and alpha suppression would work as a good manipulation check. If not, this should be discussed as a limitation of the study.

9. As an alternative manipulation check, a correlation between sensor-level results between pain condition minus neutral and the VPQ would be important to show that results at a neural level indeed index empathy for pain.

10. the lack of relationship between VPQ and source in study 1 (if I understand this correctly) needs to be better addressed at the discussion.

Conclusion:

11. the debate, starting with Oxley 2008 should be discussed since the introduction.

12. The authors seem to interpret the lack of significant differences in empathy responses between political groups as a straightforward null finding. However, it's crucial to consider that null results do not necessarily indicate the absence of an effect. They could be due to insufficient statistical power, the variability of the sample, or the sensitivity of the measures used. A more critical discussion on the power analysis and whether the study was adequately powered to detect small but potentially meaningful differences between groups would strengthen the interpretation.

13. Relatedly, please also add Bayesian statistics as this would provide information about how weak or strong is the evidence in favour for the null or alternative hypothesis.

14. To explain the discrepancy in findings with their earlier study, the authors mention that while their last study relied on perspective taking and mentalising as the stimuli involved suffering, whereas the current one depicted pain to body parts and as such engages bottom-up processing. I do not think this is the case. Ultimately, it should also involve mentalising and perspective taking, and as far as I know it relies more on top-down processing as well. There is an overlap in activation between vicarious and actual pain in the insula and rTPJ, which is also activated by empathy for suffering. Indeed, empathy for pain has been found to involve the affective but not sensory aspects of pain (e.g., see Singer et al., 2004).

15. In page 19-20, the authors mention that the neurobiology of empathy suggests different brain regions for emotional empathy and empathy for pain. Yet, the authors don't mention the brain regions found as a source in the current paper, compared to their previous study (Zebarjadi et al., 2023). Relatedly, the source of the alpha suppression, and if this in line with studies examining vicarious pain, should be discussed (while acknowledging the limitations of MEG in terms of spatial resolution).

Minor points:

16. Please expand more detailed demographic information (e.g., educational background, ethnicity, socioeconomic status) in order to offer insights into the sample's representativeness and the generalizability of the findings.

17. Lines 329 to 331, please give some stats. Also, given that what you are measuring is the mean (and not the median) with the t test, please add this and SEM in the raincloud plots in Figure 4.

18. Regarding the self-reported measures, provide more detail on each measure's crombachs alpha, means and SD for each sample

19. P.10 line 232, Please change 'insignificant' to non-significant

20. Line 255 change insignificant to non-significant

## Author Response

New Vistas for the Relationship between Empathy and Political Ideology Dear Editor, Thank you very much for considering our manuscript for publication in eNeuro. We addressed all the concerns and comments by the editor and reviewers as detailed below. Corrections in the manuscript are highlighted in yellow.

Sincerely, Niloufar Zebarjadi and Jonathan Levy Editor's comments:

- "depicting painful (P) conditions" -- Please give a brief description explaining how exactly pain is depicted on these images. Please specify how large the stimuli were in degrees of visual angle and how long they were presented.

Response: We thank the editor for pointing this out. The painful condition consists of images containing physical pain such as injuries or wounds in the body to elicit pain empathy. Stimuli were displayed randomly in a standardized size at the center of the monitor with a visual angle of 20.96{degree sign} × 15.37{degree sign} for 1 second. This detail has been added and highlighted in the Method section, Stimuli subsection for each study, pages 5 and 9.

- "Artifacts were eliminated from the raw data, using independent component analysis" -- please specify ICA algorithm and the criteria that were used to select components for rejection.

Response: We thank the editor for this comment. We used the runICA and fastICA algorithms for studies 1 and 2, respectively with the rejection threshold of 4e-12 for mag, and 4000e-13 for grad. Then, the components corresponding to artifacts were identified through visual inspection of their time courses and topographies, focusing on patterns characteristic of eye blinks and heartbeats. These now were added and highlighted in the text in the Method section, Brain data analysis subsections for each study, pages 6 and 10.

- "onsets of the stimuli were detected" -- Does this refer to conventional triggers that were synchronized with image onsets? What exactly does "detection" refer to? Please clarify.

Response: We thank the editor for this point, yes, it refers to conventional triggers. The change was applied and highlighted in the text in the Method section, Brain data analysis subsections pages 6.

- Time-frequency analysis: please specify the exact frequency range of the "alpha band".

Response: Thanks for this comment, the alpha band range (6-14 Hz) was added and highlighted in page 6.

- "the statistical contrast between the two conditions (pain versus neutral) after adjusting for multiple comparisons was calculated" -- how was the test calculated? Which method was used for adjusting for multiple testing and how was the number of tests determined (number of frequencies, time points, channels, etc.)? How does this test relate to the cluster permutation test that is mentioned later on? If I understood correctly, the cluster test was computed in channel space (not source space), as well.

Response: We thank the editor for this important comment. The test to calculate the statistical contrast between the conditions is cluster-based permutation test, as detailed in the statistical analysis subsection, regardless whether it is at the sensor or source levels. We added these details to the manuscript, statistical analysis subsections. To make it clearer, we revised the sentence and added a note to specify this connection. In addition, we added subtitles to the sensor, source and statistical analysis subsections. Changes were highlighted in pages 7.

Regarding the number of tests, for all sensor analysis, the number of frequencies, timepoints and channels are 9, 41 and 1 (averaged over the channels), respectively. For the source analysis, the number of frequencies, timepoints and channels for the first study are 5, 6 and 1, and for the second study are 5,9 and 1, respectively. We added and highlighted these details to the manuscript, result section for the whole sample subsection at page 11, for study 1 section at page 13, and for study 2 section at pages 14 and 15.

- "Initially, t-values, indicating the differences between the conditions, were computed for each subject, channel, frequency, and time... The results were corrected for multiple comparisons along the time and frequency dimensions" -- The correction should also account for the channel dimension.

Response: Thanks for this point, the correction was applied and highlighted in page 7.

- MEG data analysis is only reported for Study 1. Were Study 2 data processed in exactly the same way? Please clarify.

Response: We thank the editor for pointing this out. The brain data analysis for the two studies is almost identical to compare the results of the two studies. There are only minor differences due to technical/system-related reasons, that were mentioned in the Brain data analysis subsection for study 2 in page 10. The sentence mentioning the same analysis for both studies was revised and highlighted in the same subsection.

- "The sensor analysis of all 124 subjects determined a significant alpha power suppression pattern".

The MEG results are often referred to as "alpha power suppression" or "a significant decrease in alpha power." If I understand correctly, this does not refer to changes in alpha power pre se (i.e. with respect to the pre-stimulus interval), but to differences between the two image categories. I think this should be stated explicitly, at least when such results are mentioned for the first time. Moreover, I think this would help explain the polarity of this effect. I suppose that the stimulus onset induces desynchronization under both conditions. Which condition desynchronizes more strongly when the t-value is negative? Response: We thank the editor for this comment. Yes, the suppression is between the two conditions and not alpha power per se and the greater desynchronization occurs for the pain condition. We now added and highlighted this point in the results section for the whole sample and each study separately on pages 11, 12, and 14, respectively.

- I think the rationale for focusing on this particular frequency band should be explained in more detail. How was this frequency band determined a priori to be the frequency range of interest? Moreover, the discussion of these results should be expanded. Specifically, how is alpha power thought to be related to empathy, and why? In the figures, the MEG effect is referred to as "pain empathy neural response," which seems to refer to a strong interpretation of what this signal reflects. What exactly is the evidence that alpha suppression is a correlate of *empathy* for the person depicted in these images rather than a correlate of something else, such as attention, interest, arousal, or physical characteristics of these images? How does the tentative role of the alpha rhythm for empathy jibe with the vast literature on the connection between alpha and arousal and attention? Response: We thank the editor for this point. We added and highlighted more information in the last paragraph of the introduction (page 4-5) and third paragraph of discussion ( page 21) regarding the role of alpha suppression in empathy response.

Reviewer's comments:

- While previous research by the authors (Zebarjadi et al., 2023) showed that there is ideological asymmetry in cognitive-affective empathy, the authors now show that there is no evidence for ideological assymetry in empathy for pain. Even though I find this interesting, I am not entirely convinced about the author's rational of proposing a distinction in empathy type between this and their last study given that empathy for pain also involves affective, cognitive aspects of empathy and there is mentalisation in both cases (i.e., in response to distressing stories or seeing people in pain). I also question their justification of these being separate processes because "empathy for pain relies on bottom up processes".

Response: We thank the reviewer for this comment.

We agree with the reviewer that pain empathy involves several processes (as we claimed in the Zebarjadi et al 2021 article), however, the processes in the "mental" paradigm used in zebarjadi et al 2023 uses a much more affective and mentalizing based processes - the paradigm is based on stories and pictures of people in emotional situations, and the instruction to mentalize about the stories and state they are found in. This article indeed indicated a TPJ activation - a typical mentalization/TOM hub. Therefore, although topdown processes is included in the "physical pain empathy paradigm", the activations rely far less on affective and mentalizing processes compared to the "mental paradigm" in 2023.

In addition, regarding the engagement of various empathy components depending on the stimuli eliciting empathic responses, several former studies indicated this point (Bruneau et al., 2012; Lamm et al., 2011). For instance, the meta-analysis by Lamm et al. (2011) highlighted distinct brain regions activated during empathy depending on the type of experimental paradigm. They discussed that images of body parts in painful situations activate neural structures involved in understanding and predicting the outcomes of these situations, thereby triggering inferences about their affective consequences. In contrast, stimuli associated with TOM elicit inferences about self- and other-related social information and facilitate the sharing of other's state based on one's own prior experiences and knowledge (Lamm et al., 2011).

We removed "engages bottom-up processing" to avoid confusion, and added and highlighted more description about former studies that discussed this distinction in the 4th paragraph of Discussion at page 23.

- I also ask the authors to provide more details about their "political ideology measure". This seems to involve a 7 point likert scale but there is no mention of how where participants assigned to leftists vs rightists (e.g., median split?). Details about how/if it was validated, and whether it adequately captures the complexity of political ideology are also needed as examining differences between rightists and leftists is the main aim of the paper.

Response: We thank the reviewer for pointing out this important point. We now explained and highlighted the details as follow at self-reported measures section at page10:

In this self-reported measure, participants who rated 1-3 on the political ideology scale, explicitly reported that they are rightists (extreme, medium, light), and therefore, we classified them as the rightists, and those who rated 5-7, explicitly reported that they are leftists (light, medium, extreme), and therefore, we classified as the leftists. For the Israeli dataset, we assessed the subjects' reading habits by asking them to rate their preferences for two national newspapers in Israel: the left-leaning Ha'aretz and the right-leaning Israel Hayom on a scale from 1 (not at all) to 7 (all the time). As has been implemented in our previous work (Zebarjadi et al., 2023), these reading habit ratings were used first to verify the results of the political ideology scale and second to assign those who scored 4 to either leftist or rightist group. No Inconsistency was found between the results of the political ideology and reading habit scales. For the Finnish dataset, the validation of the political ideology scale and categorization of those who scored 4 were done based on an initial online survey using the right-wing authoritarian (RWA) scale.

Statistics:

The statistical analyses are not clearly explained in some sections and therefore I cannot judge if they are correct or not.

In some other parts it seems that the authors analyzed the pain vs neutral conditions and then the leftist vs rightist on alpha suppression. Given they have a 2 x 2 design ideally they should analyse them in a way to look for an interaction (e.g., 2 x 2 anova). It is also not clear if the ideological asymmetry was only analyzed in the pain condition (or on both).

Response: We thank the reviewer for this valuable feedback. We appreciate the suggestion to consider a 2x2 ANOVA approach. However, in MEG analysis of empathy paradigms, the primary focus is on the pain vs. no-pain contrast because it specifically reflects alpha suppression, which we use as an estimation of the empathy response in the brain. Analyzing the two stimulus conditions separately would not capture the neural processes associated with pain empathy, as each condition involves various unrelated processes (e.g., visual processing). Therefore, the contrast between pain and no-pain is critical to isolating the pain empathy process. This approach is very common in MEG studies examining the neural oscillatory underpinnings of empathy (Levy et al., 2016, 2018, 2019; Whitmarsh et al., 2011; Zebarjadi et al., 2021; 2023), and the latter neural representation is typically used using the non-parametric cluster-based permutation statistical approach (Marris &Oostenveld, 2007), and can straightforwardly be applied, for instance, by comparing the representations in each of the two groups (i.e., rightists vs leftists).

This information is now further emphasized on page 7 in the highlighted text of the "Statistical analysis".

The authors seem to interpret the lack of significant differences in empathy responses between political groups as a straightforward null finding. However, it's crucial to consider that null results do not necessarily indicate the absence of an effect. Further analyses are needed: for example, Bayes factor to provide information about how weak or strong is the evidence in favour for the null or alternative hypothesis.

Response: We thank the reviewer for pointing out this issue and suggesting a statistical way to back our claim. We computed Bayesian factors for each of the analyses. The BFs were all smaller than one and ranged from 0.19 to 0.75, suggesting anecdotal to moderate support for the H0 hypotheses. Thus, although the results are not unequivocally, the consistency of the results supports our claim that there is no political differences in this task. We addressed this point in the manuscript and we highlighted it in the method (page 8), result (pages 12,13,14 and 15), and discussion (page 22) .

Evaluation:

Introduction 1. I am not entirely convinced about the author's rational proposing a distinction in empathy type between this and your last study (Zebarjadi et al., 2023) given that empathy for pain also involves affective, cognitive aspects of empathy and there is mentalisation in both cases (i.e., in response to distressing stories or seeing people in pain) Response: We thank the reviewer for this comment.

We agree with the reviewer that pain empathy involves several processes (as we claimed in the Zebarjadi et al 2021 article), however, the processes in the "mental" paradigm used in zebarjadi et al 2023 uses a much more affective and mentalizing based processes - the paradigm is based on stories and pictures of people in emotional situations, and the instruction to mentalize about the stories and state they are found in. This article indeed indicated a TPJ activation - a typical mentalization/TOM hub. Therefore, although top-down processes is included in the "physical pain empathy paradigm", the activations rely far less on affective and mentalizing processes compared to the "mental paradigm" in 2023.

In addition, regarding the engagement of various empathy components depending on the stimuli eliciting empathic responses, several former studies indicated this point (Bruneau et al., 2012; Lamm et al., 2011). For instance, the meta-analysis by Lamm et al. (2011) highlighted distinct brain regions activated during empathy depending on the type of experimental paradigm. They discussed that images of body parts in painful situations activate neural structures involved in understanding and predicting the outcomes of these situations, thereby triggering inferences about their affective consequences. In contrast, stimuli associated with TOM elicit inferences about self- and other-related social information and facilitate the sharing of other's state based on one's own prior experiences and knowledge (Lamm et al., 2011).

We removed "engages bottom-up processing" to avoid confusion, and added and highlighted more explanation in the 4th paragraph of Discussion at page 23.

Methods 2. The method used to classify participants as politically left or right is briefly mentioned, involving a seven-point scale, and this needs to be better approached. For example, where participants assigned as leftists or rightists by doing a median split, or by only taking the extremes? The manuscript could also benefit from a more detailed description of this scale, e.g., how it was validated, and whether it adequately captures the complexity of political ideology. This is crucial as examining differences between these is the main aim of the paper.

Response: We thank the reviewer for pointing out this important point. We now explained and highlighted the details as follow at self-reported measures section at page 10 :

In this self-reported measure, participants who rated 1-3 on the political ideology scale, explicitly reported that they are rightists (extreme, medium, light), and therefore, we classified them as the rightists, and those who rated 5-7, explicitly reported that they are leftists (light, medium, extreme), and therefore, we classified as the leftists. For the Israeli dataset, we assessed the subjects' reading habits by asking them to rate their preferences for two national newspapers in Israel: the left-leaning Ha'aretz and the right-leaning Israel Hayom on a scale from 1 (not at all) to 7 (all the time). As has been implemented in our previous work (Zebarjadi et al., 2023), these reading habit ratings were used first to verify the results of the political ideology scale and second to assign those who scored 4 to either leftist or rightist group. No Inconsistency was found between the results of the political ideology and reading habit scales. For the Finnish dataset, the validation of the political ideology scale and categorization of those who scored 4 were done based on an initial online survey using the right-wing authoritarian (RWA) scale.

Study 1 results 3. Overall, results need to be explained better. For example, explain what this means for non-technical audience: " Whole-brain sensor analysis on 77 participants within the alpha range, shown in Figure 2A, revealed a significant suppression in alpha power (negative Pcluster-cor < 0.001, T= -5.4, permutation test).

Response: We thank the reviewer for this comment. This significant alpha suppression at the sensor level during physical pain empathy task may imply a possible pain empathy effect in this population. We added and highlighted explanation at page 13 for this sentence to make it clearer.

4. The authors report no changes in alpha suppression between rightists and leftists (t test), but a more nuanced approach would be a better fit to examine whether the results are specific for empathy for pain (relative to neutral) and differ by political ideology. This should include all conditions and interactions, e.g., a 2 (stimuli: neutral, painful stimuli ) x 2( ideology : rightist , leftist) anova. An ideal outcome would be a significant interaction effect, indicating that the empathetic response to pain (as reflected in alpha suppression) is not only specific to painful stimuli but also varies according to political ideology. Yet if non-parametric tests are necessary due to data distribution, then the authors could show this by, for example, looking at difference scores (although not ideal).

Response: We thank again the reviewer for raising this point, which we elaborated over in the answer to the question relating to Statistics (c.f., page 5 in this response letter).

Study 2 Results 5. Results need to be written more clearly and explain what they suggest taken together.

Response: We thank the reviewer for this comment. Similar to Study 1, this significant alpha suppression may indicate an effect in this population in response to pain empathy stimuli. We added and highlighted explanation at page 14 for this sentence to make it clearer.

6. If I understand correctly, there are two comparisons done separately at a source level : one comparing suppression between neutral and pain and another rightists between leftists. However, to investigate the research question, one would expect a difference between leftists and rightists in pain vs neutral conditions, and unless I am missing something, this isn't being tested here. See also point above in relation to study 1.

Response: We thank the reviewer for raising this point. Building on our previous response to the question relating to Statistics (c.f., page 5 in this response letter), the empathy neural representation is calculated using the contrast of pain vs no-pain, on which the non-parametric permutation procedure is applied regardless of sensor or source. Then, as typically applied (Levy et al., 2016, 2018, 2019; Whitmarsh et al., 2011; Zebarjadi et al., 2021; 2023), for sensor space the permutation procedure is applied on the time-frequency-sensor dimensions (e.g., figure 2A), whereas in source space it is on the time dimension (e.g., figure 2E). The outputted representation is then compared between the two groups of interest in sensor (e.g., figure 2C) and in source (e.g., figure 2F).

Behavioral analyses 7. Almost the whole entire first paragraph needs to be re-written (lines 317-326). For example, what do the VPQ subscales measure? This hasn't been explained earlier so we cannot follow. Or do the authors mean scale instead of subscale ? And the authors mention that they are correlating questionnaires to sensor-level results, but in response to what experimental condition? Also, Line 321 to 324 is not clear. And Line 324, 325, 326 needs to be better explained on how you got to that conclusion.

Response: We thank the reviewer for this point. We now added a new paragraph in the beginning to provide an initial explanation and revised the other paragraph to make it clearer. We highlighted the changes at behavioral analysis section, page 17-18.

8. Did the authors collect vicarious pain ratings as a behavioural measure of pain in response to each picture? Correlational analyses between these and alpha suppression would work as a good manipulation check. If not, this should be discussed as a limitation of the study.

Response: We thank the reviewer for this comment.

For the Finnish dataset, we collected the pain ratings and we now evaluated the Pearson correlation between alpha suppression at the peak coordinate and the self-reported ratings of pain stimuli and the correlation was significant (r= -0.3, p= 0.44). We also calculated the contrast between the two political groups for this metric and it was not significant (p=0.22). this information were added and highlighted in behavioral analysis section, paragraph 2 and 3, in page 18.

For Israeli dataset, the pain rating has not been collected and we added and highlighted this point as a limitation, discussion section, paragraph 4, at page 24, as follow:

In this study, self-reported vicarious pain ratings has not been measured for the Israeli dataset and are suggested to be incorporated in future research to provide further correlational analysis between the neural findings and self-reported pain response to the same stimuli.

9. As an alternative manipulation check, a correlation between sensor-level results between pain condition minus neutral and the VPQ would be important to show that results at a neural level indeed index empathy for pain.

Response: We thank the reviewer for this comment. The correlational check between VPQ results (both pain intensity and discomfort measures) and the sensor-level neural results (indicating the contrast between pain and neutral conditions at sensor level) has been done in the behavioral analysis section (second paragraph) at page 17-18 which has been now re-written and highlighted to better explain the manipulation check.

10. the lack of relationship between VPQ and source in study 1 (if I understand this correctly) needs to be better addressed at the discussion.

Response: We thank the reviewer for this point. lack of relationship between VPQ and source has been in both studies. We now discussed more the neural-behavioral correlation, both on the detected sensor-VPQ correlation and lack of source-behavioral correlation, in the discussion section, paragraph 4, at page 24 (changes are highlighted), as follow:

Furthermore, Neural-behavioral analysis indicated a weak correlation between sensor neural results and one of the VPQ measures, whereas non-significant correlations were found between this neural finding and IRI measures. This may suggest that the neural measures are more closely associated with behavioral real-life sensitivity to vicarious pain rather than with self-reported trait empathy. However, no significant correlation was found between the source neural findings and behavioral or self-reported measures which might be due to the limitations imposed by the MEG measurement or lower sample size compared to the psychological studies, as reported in the previous research (DiGirolamo et al., 2019; Whitmarsh et al., 2011).

Conclusion:

11. the debate, starting with Oxley 2008 should be discussed since the introduction.

Response: We thank the reviewer for this comment, we now added the debate in the introduction as well. The text we highlighted in introduction, paragraph 3 page 4 .

12. The authors seem to interpret the lack of significant differences in empathy responses between political groups as a straightforward null finding. However, it's crucial to consider that null results do not necessarily indicate the absence of an effect. They could be due to insufficient statistical power, the variability of the sample, or the sensitivity of the measures used. A more critical discussion on the power analysis and whether the study was adequately powered to detect small but potentially meaningful differences between groups would strengthen the interpretation.

13. Relatedly, please also add Bayesian statistics as this would provide information about how weak or strong is the evidence in favour for the null or alternative hypothesis.

Response: We thank the reviewer for raising these two points, and for suggesting a statistical way to further examine the reported data.

We computed Bayesian factors for each of the analyses. The BFs were all smaller than one and ranged from 0.19 to 0.75, suggesting anecdotal to moderate support for the H0 hypotheses. Thus, although the results are not unequivocally, the consistency of the results supports our claim that there is no political differences in this task. We addressed this point in the manuscript and we highlighted it in the method (page 8), result (pages 12,13,14 and 15), and discussion (page 22). In addition, in the Discussion we emphasized that the detected null results in the current study might not necessarily indicate the absence of difference between the political groups (highlighted).

14. To explain the discrepancy in findings with their earlier study, the authors mention that while their last study relied on perspective taking and mentalising as the stimuli involved suffering, whereas the current one depicted pain to body parts and as such engages bottom-up processing. I do not think this is the case. Ultimately, it should also involve mentalising and perspective taking, and as far as I know it relies more on top-down processing as well. There is an overlap in activation between vicarious and actual pain in the insula and rTPJ, which is also activated by empathy for suffering. Indeed, empathy for pain has been found to involve the affective but not sensory aspects of pain (e.g., see Singer et al., 2004).

Response: We thank the reviewer for this comment.

We agree with the reviewer that pain empathy involves several processes (as we claimed in the Zebarjadi et al 2021 article), however, the processes in the "mental" paradigm used in Zebarjadi et al., 2023 uses a much more affective and mentalizing based processes - the paradigm is based on stories and pictures of people in emotional situations, and the instruction to mentalize about the stories and state they are found in. This article indeed indicated a TPJ activation - a typical mentalization/TOM hub. Therefore, although topdown processes is included in the "physical pain empathy paradigm", the activations rely far less on affective and mentalizing processes compared to the "mental paradigm" in 2023.

In addition, regarding the engagement of various empathy components depending on the stimuli eliciting empathic responses, several former studies indicated this point (Bruneau et al., 2012; Lamm et al., 2011). For instance, the meta-analysis by Lamm et al. (2011) highlighted distinct brain regions activated during empathy depending on the type of experimental paradigm. They discussed that images of body parts in painful situations activate neural structures involved in understanding and predicting the outcomes of these situations, thereby triggering inferences about their affective consequences. In contrast, stimuli associated with TOM elicit inferences about self- and other-related social information and facilitate the sharing of other's state based on one's own prior experiences and knowledge (Lamm et al., 2011).

We removed "engages bottom-up processing" to avoid confusion, and added and highlighted more description about former studies that discussed this distinction in the 4th paragraph of Discussion at page 23.

15. In page 19-20, the authors mention that the neurobiology of empathy suggests different brain regions for emotional empathy and empathy for pain. Yet, the authors don't mention the brain regions found as a source in the current paper, compared to their previous study (Zebarjadi et al., 2023). Relatedly, the source of the alpha suppression, and if this in line with studies examining vicarious pain, should be discussed (while acknowledging the limitations of MEG in terms of spatial resolution).

Response: We thank the reviewer for this important point. We elaborated more on this point in the discussion. Changes were highlighted in the third paragraph of discussion in pages 21-22.

Minor points:

16. Please expand more detailed demographic information (e.g., educational background, ethnicity, socioeconomic status) in order to offer insights into the sample's representativeness and the generalizability of the findings.

Response: We thank the reviewer for this suggestion. We now added and highlighted the ethnicity of the participants to the Method, participants subsection at pages 5 and 8 for each study. Regarding other demographics such as education or socioeconomic status, unfortunately we have not collected this information through the current study, which is considered in future research.

17. Lines 329 to 331, please give some stats. Also, given that what you are measuring is the mean (and not the median) with the t test, please add this and SEM in the raincloud plots in Figure 4.

Response: We thank the reviewer for this comment. we added and highlighted stats, mean and SEM as follows in the behavioral analysis section at page 18:

VPQ (VPQ1: p= 0.15, mean= 2.33, SEM= 0.21; VPQ2: p= 0.10, mean= 4.66, SEM= 0.19) and IRI (IRI(EC): p= 0.08, mean= 5.8, SEM= 0.08; (IRI(PT): p= 0.13, mean= 5.6, SEM= 0.08) 18. Regarding the self-reported measures, provide more detail on each measure's crombachs alpha, means and SD for each sample Response: We thank the reviewer for this important point. We now provided and highlighted these details for each measure in each sample in Table 1, behavioral analysis section, page 17.

19 and 20. P.10 line 232, Please change 'insignificant' to non-significant and Line 255 change insignificant to non-significant Response: We thank the reviewer for this point, the corrections from 'insignificant' to 'non-significant' are applied and highlighted through the text at pages 12,13,15, 16 and 17.

References:

Bruneau, E. G., Pluta, A., &Saxe, R. (2012). Distinct roles of the 'Shared Pain' and 'Theory of Mind' networks in processing others' emotional suffering. Neuropsychologia, 50(2), 219-231. https://doi.org/10.1016/J.NEUROPSYCHOLOGIA.2011.11.008 DiGirolamo, M. A., Simon, J. C., Hubley, K. M., Kopulsky, A., &Gutsell, J. N. (2019). Clarifying the relationship between trait empathy and action-based resonance indexed by EEG mu-rhythm suppression. Neuropsychologia, 133, 107172. https://doi.org/10.1016/j.neuropsychologia.2019.107172 Lamm, C., Decety, J., &Singer, T. (2011). Meta-analytic evidence for common and distinct neural networks associated with directly experienced pain and empathy for pain. Neuroimage, 54(3), 2492-2502.

Levy, J., Goldstein, A., &Feldman, R. (2019). The neural development of empathy is sensitive to caregiving and early trauma. Nature Communications, 10(1), 1-10.

Levy, J., Goldstein, A., Influs, M., Masalha, S., Zagoory-Sharon, O., &Feldman, R. (2016). Adolescents growing up amidst intractable conflict attenuate brain response to pain of outgroup. Proceedings of the National Academy of Sciences, 113(48), 13696-13701.

Levy, J., Goldstein, A., Pratt, M., &Feldman, R. (2018). Maturation of pain empathy from child to adult shifts from single to multiple neural rhythms to support interoceptive representations. Scientific Reports, 8(1), 1-9.

Maris E, O. R. (2007). Nonparametric statistical testing of EEG- and MEG-data. J Neurosci Methods., 164(1), 177-190.

Whitmarsh, S., Nieuwenhuis, I. L. C., Barendregt, H., &Jensen, O. (2011). Sensorimotor alpha activity is modulated in response to the observation of pain in others. Frontiers in Human Neuroscience, 5, 91.

Zebarjadi, N., Adler, E., Kluge, A., Sams, M., &Levy, J. (2023). Ideological values are parametrically associated with empathy neural response to vicarious suffering. Social Cognitive and Affective Neuroscience, 18(1), nsad029. https://doi.org/10.1093/scan/nsad029 Zebarjadi, N., Adler, E., Kluge, A., Jääskeläinen, I. P., Sams, M., &Levy, J. (2021). Rhythmic Neural Patterns During Empathy to Vicarious Pain: Beyond the Affective-Cognitive Empathy Dichotomy . In Frontiers in Human Neuroscience (Vol. 15, p. 380).
